# High Illuminance and Low Temperature Promote Perithecial Production of the Green Bristlegrass Blast Fungus *Magnaporthe oryzae*

**DOI:** 10.3390/jof12080564

**Published:** 2026-08-01

**Authors:** Jintao Liu, Zixue Wu, Huiying Li, Qifeng Zhang, Haixu Sang, Jun Yang

**Affiliations:** 1 State Key Laboratory of Agricultural and Forestry Biosecurity, MARA Key Laboratory of Surveillance and Management for Plant Quarantine Pests, College of Plant Protection, China Agricultural University, Beijing 100193, China; liujt0609@163.com (J.L.);; 2 Liaoning Province Saline-Alkali Land Utilization Research Institute, Panjin 124010, China; 3 Heilongjiang Provincial Plant Inspection and Quarantine Station, Harbin 150036, China

**Keywords:** blast fungus, perithecia, sexual reproduction, environmental factor

## Abstract

Sexual reproduction of the ascomycetous fungus *Magnaporthe oryzae* is reported to be regulated by illuminance and temperature, but their temporal interaction during perithecial production remains unclear. Here, we performed full-factorial condition transfer assays (illuminance: low/0 lx, medium/1000 lx, or high/5000 lx; temperature: low/19 °C or high/25 °C) using a pair of high-fertility *M. oryzae* isolates collected from green bristlegrass to characterize sequential and combinatorial effects of illuminance and temperature on perithecial production. We found that perithecial production showed a strong positive correlation with illuminance at 19 °C, and continuous darkness completely abolished their fertility. Early illuminance priming promotes perithecial production in a dose-dependent manner, whereas prolonged initial darkness for over four days results in remarkable reductions in perithecial production. Moreover, pre-transfer at 25 °C promoted perithecial production under 1000 lx or darkness, and post-transfer at 19 °C sustained or enhanced perithecial production. We also found that plates initially cultured at 5000 lx maintain high capacity for perithecial production across most post-transfer environments, especially under the post-transfer condition at 19 °C with over 2000 perithecia per plate. Taken together, our study demonstrates that combinations of high illuminance and low temperature promote perithecial production of the green bristlegrass blast fungus *M. oryzae* under laboratory conditions, providing alternative insights into the roles of environmental factors in fungal sexual reproduction.

## 1. Introduction

Genetic recombination via sexual reproduction underpins one of the most powerful approaches for analyzing genetic variation in fungi [1]. To fully exploit this method, reliable induction of the sexual stage through controlled mating of selected strains is essential. While environmental regulators of fungal sexual development have been extensively documented, optimal illuminance and temperature requirements vary dramatically across species [2]. Light can positively impact fruiting body development by influencing the initiation and direction of growth; however, some species may require darkness or specific illuminance conditions to thrive [3]. Depending on the fruiting body species, the optimum illuminance for the fruiting body ranges from near darkness (0  lx) to moderately high levels (10–2000  lx) [4]. Similarly, temperature requirements are highly species-specific. *Podosphaera aphanis* exhibits a dose-dependent increase in chasmothecial initiation upon prolonged exposure to 13 °C compared with constant 25 °C incubation [5]. In *Fusarium graminearum*, perithecia are produced between 5 °C and 30 °C (optimum at 21.7 °C) but mature only at 20 °C and 25 °C [6].

*Magnaporthe oryzae* is a devastating hemibiotrophic fungal pathogen with an extremely broad host range, infecting more than 50 species of gramineous crops and weeds, including rice (*Oryza sativa*), foxtail millet (*Setaria italica*), and green bristlegrass (*S. viridis*) [7,8]. It has also emerged as a global threat to wheat (*Triticum aestivum*) production following the evolution of host-shifted isolates that overcome non-host resistance to cause wheat blast [9]. Sexual reproduction is a major driver of adaptive evolution in *M. oryzae*: through meiosis and genetic recombination, it generates novel genotypes with enhanced genetic diversity, which facilitates host range expansion, breakdown of plant resistance, and adaptation to changing environments [10,11,12,13,14]. Since Hebert first reported the sexual stage of *M. grisea* under laboratory conditions in 1971 [15], subsequent studies have confirmed that *M. oryzae* isolates from rice, foxtail millet, and green bristlegrass are all capable of producing perithecia on artificial media [16,17]. Nevertheless, most previous studies relied on constant culture conditions [17,18,19,20], and the dynamic, stage-specific requirements for illuminance and temperature during sexual reproduction remain uncharacterized. Therefore, elucidating the roles of illuminance and temperature in perithecial production will contribute to the investigation of sexual reproduction in *M. oryzae*.

In this study, we used a pair of sexually compatible *M. oryzae* isolates, G10a and G10b, collected from naturally infected green bristlegrass in Beijing, China. By performing full-factorial condition-transfer assays with three illuminance levels and two temperature parameters at different stages of sexual development, we quantified perithecial production across 36 treatments, investigated the interactive effects of illuminance and temperature on perithecial production, and determined the highest-yielding candidate condition for perithecial production.

## 2. Materials and Methods

### 2.1. Fungal Isolates and Culture Conditions

Ten monoconidial isolates were isolated from diseased leaves of blast-infected green bristlegrass collected in Beijing, China (116.2824° E, 40.0275° N). Among random paired crossings under a routine approach, the G10a × G10b pair exhibited the highest fertility and was selected for further analysis (Figure S1A). The mating types of G10a and G10b were determined via PCR by using mating type-specific primers [21], and G10a was *MAT1-1* and G10b was *MAT1-2* (Figure S1B). Fungal strains were maintained on oatmeal tomato agar (OTA; 150 mL tomato juice, 20 g oatmeal, and 15 g agar per liter) plates at 25 °C and stored as mycelia on sterilized filter paper at −20 °C for long-term preservation. The incubators (GXZ-308C-LED, Ningbo Jiangnan Instrument Factory, Ningbo, China) were equipped with LED light sources (color temperature 5500 K). Light treatments were set by adjusting LED brightness to 100% (5000 lx), 20% (1000 lx), and 0% (0 lx), and were applied as continuous 24 h. Plates were placed 20 cm from the lamps, and the illuminance was calibrated with a portable light meter (FT3424, HIOKI, Ueda City, Japan) held just above the plate surface. Plates were rotated 180° daily to ensure uniform light exposure.

### 2.2. Condition-Transferred Sexual Crossing Assays

**Figure 1:**
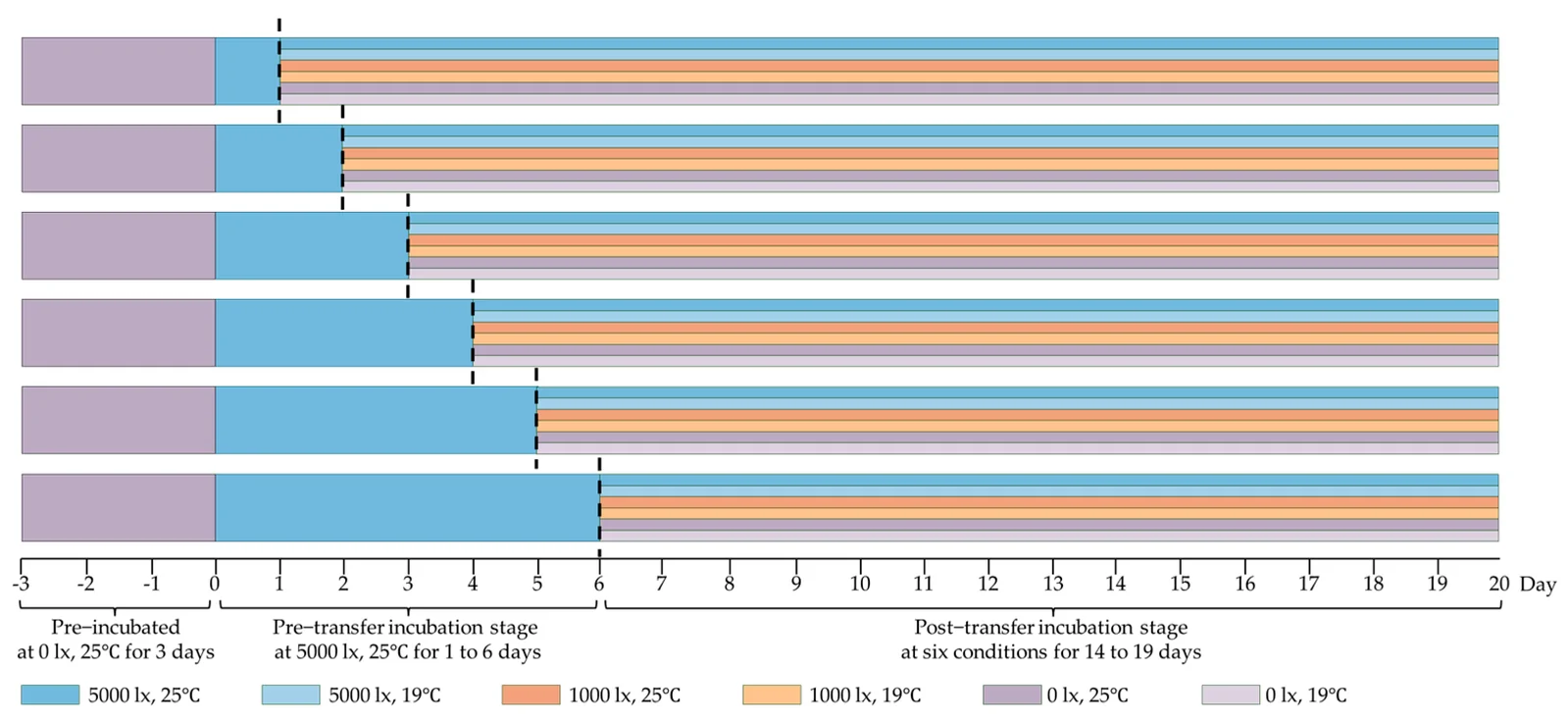
Schematic timeline of the environment transfer assay (representative example: the pre-transfer environment of 5000 lx, 25 °C). Plates were pre-incubated in 0 lx, 25 °C for 3 days (dusty purple segment), and were then transferred to the pre-transfer environment of 5000 lx, 25 °C and cultured for 1 to 6 d (light blue segment). The six horizontal rows correspond to six independent transfer time points, with dashed lines indicating the daily transfer performed on day 1 through day 6 of the pre-transfer stage. Following the pre-transfer incubation, plates were either maintained in their original environment or transferred to the other five post-transfer environments, comprising distinct illuminance-temperature combinations indicated by different colors: light blue (5000 lx, 25 °C), pale blue (5000 lx, 19 °C), tan (1000 lx, 25 °C), apricot (1000 lx, 19 °C), dusty purple (0 lx, 25 °C), and pale purple (0 lx, 19 °C). These plates were subsequently maintained for an additional 14 to 19 d (depending on the transfer day) for perithecial production.

Sexual crossing assays were performed by using the cross-culture method as described [19,21]. Each plate was inoculated with four mycelial plugs (5 mm), two of G10a and two of G10b, arranged in a square pattern. Plugs of opposite mating types were placed adjacent, with a center-to-center distance of 2 cm between adjacent opposite-mating-type plugs. A total of 744 plates were pre-incubated in complete darkness (0 lx) at 25 °C for 3 d, and this day was defined as day 0. When the hyphae of G10a and G10b on the plates began to come into contact, the plates were transferred to six initial environments (pre-transfer incubation stage): (1) 5000 lx, 25 °C; (2) 5000 lx, 19 °C; (3) 1000 lx, 25 °C; (4) 1000 lx, 19 °C; (5) 0 lx, 25 °C; and (6) 0 lx, 19 °C. For each initial environment, 124 plates were used. From each of the six initial environments, 24 plates were randomly selected daily, of which 4 were kept in the original environment, and the other 20 were plated equally across the other five conditions (4 plates per condition). This daily transfer procedure was repeated for six consecutive days. After transfer, the plates were maintained in new environments for an additional 14 to 19 d (post-transfer incubation stage), so that the total incubation period for the sexual stage was 20 d. The schematic timeline of the daily transfer workflow is illustrated in Figure 1, using plates under initial environments of 5000 lx and 25 °C as a representative example; the same schedule was applied to the remaining five initial environments.

### 2.3. Perithecial Quantification

After being cultured in the post-transfer environment, the number of perithecia per OTA plate was counted under a stereomicroscope (SMZ800N, Nikon, Mito, Japan) on the 20th day. Perithecia were identified as black, globose structures with protruding ostioles formed at four of the hyphal mating zones between strains G10a and G10b, and the total number of perithecia in each plate was recorded (Figure S2A–C).

### 2.4. Statistical Analysis

All statistical analyses were performed using SPSS 24.0 software, and figures were generated using GraphPad Prism 9.0. Perithecial yields are presented as mean ± SD (standard deviation) across four biological replicates per treatment. For comparisons among treatments, the Mann–Whitney U test (Wilcoxon rank-sum test) was used. The significance threshold was set at *p* < 0.05, and significance levels are marked in figures as follows: ns, not significant (*p* > 0.05); *, *p* < 0.05.

## 3. Results

### 3.1. Effects of Constant Illuminance and Temperature on Perithecial Production

**Figure 2:**
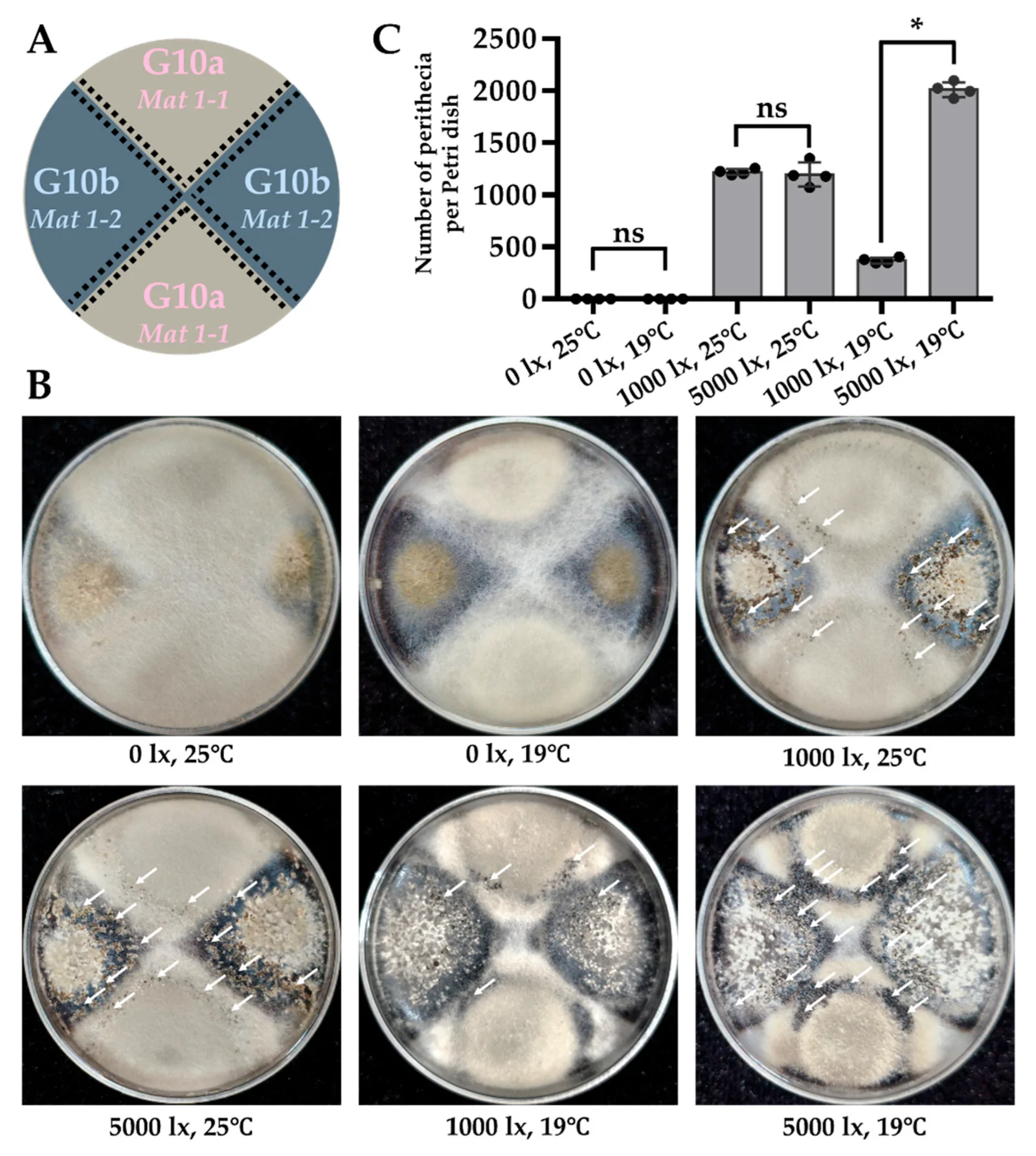
Effects of constant illuminance and temperature on perithecial production of the G10a × G10b cross. (**A**) Schematic of the G10a × G10b cross-culture on an OTA plate. (**B**) Representative images showing perithecial production under six constant illuminance-temperature conditions. White arrows indicate perithecia. (**C**) Yields of perithecia per OTA plate. The data are presented as mean ± SD from four biological replicates. Statistical significance determined by the Mann–Whitney U test (Wilcoxon rank-sum test); ns, not significant (*p* > 0.05); *, *p* < 0.05.

We firstly quantified perithecial yields under six culture conditions (Figure 2). After a 3 d pre-incubation in darkness (0 lx) at 25 °C, the plates were cultured at six constant illuminance-temperature combinations for an additional 20 days. No perithecia were detected under continuous darkness (0 lx) at either 25 °C or 19 °C, demonstrating that complete illuminance deprivation abolished perithecial production in the G10a × G10b cross, regardless of the environmental temperature. At a constant 25 °C, mean yields of perithecia were 1220 at 1000 lx and 1197 at 5000 lx. In contrast, at a constant 19 °C, mean yields of perithecia were 371 at 1000 lx but increased dramatically to 2011 at 5000 lx. To assess perithecial maturity, we randomly selected ten perithecia from plates grown under 5000 lx, which had been incubated at 19 °C for 20 d, and all of them contained abundant asci. 59.3% of the released ascospores (178 out of 300) germinated, and 98.0% of the germinated ascospores (98 out of 100) formed colonies on OTA plates (Figure S2D–G). These results indicate that fertility showed a positive correlation with increasing illuminance at 19 °C, whereas illuminance had no significant effect on fertility at 25 °C.

### 3.2. Early Illuminance Priming Promotes Perithecial Production in a Dose-Dependent Manner

**Figure 3:**
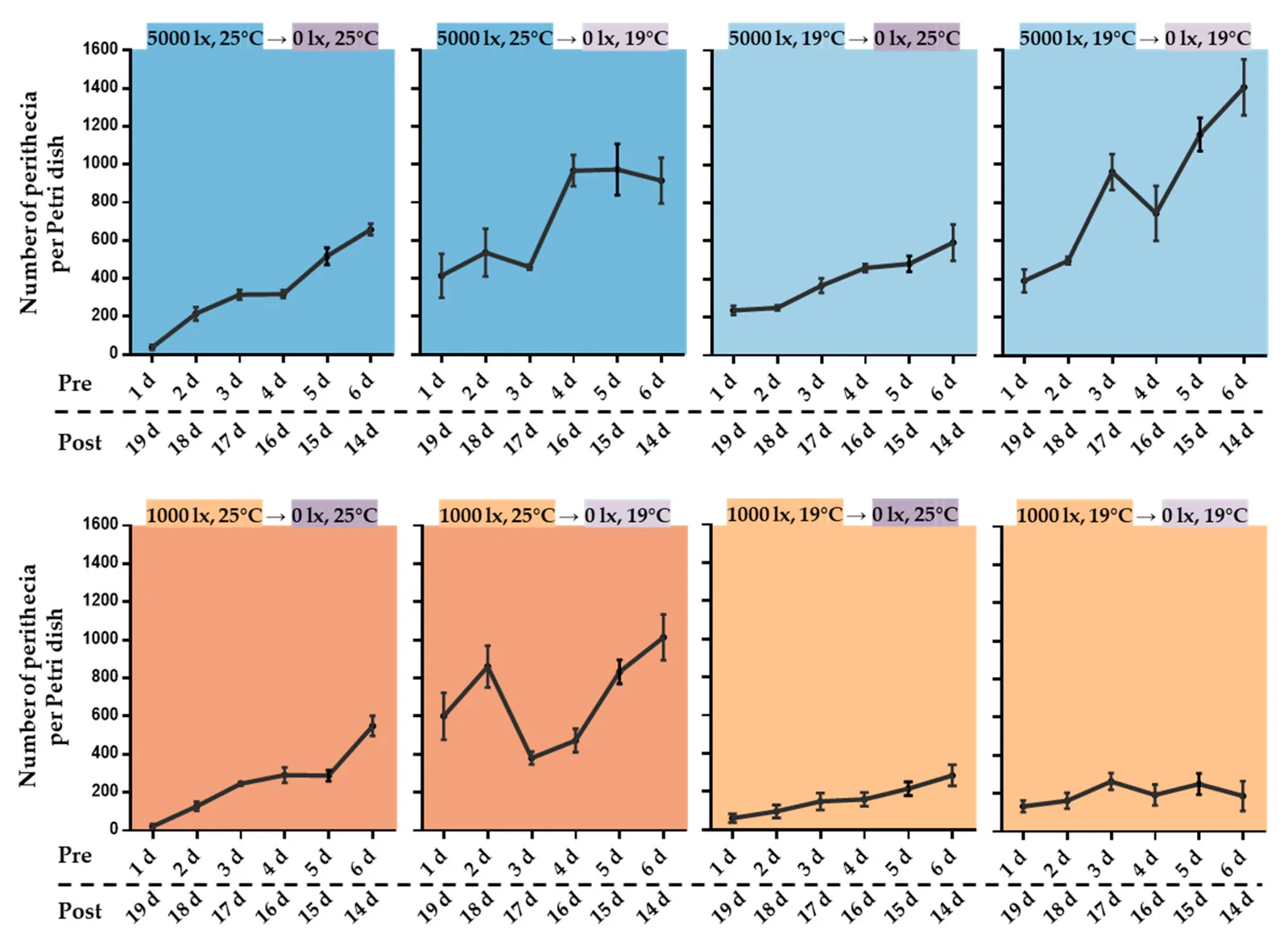
Dose-dependent effects of initial illuminance priming on perithecial production in sequential light-to-dark transfer assays. During the sexual stage, the plates were cultured under initial illumination conditions of 5000 lx (**upper row**) or 1000 lx (**lower row**), at two temperatures (25 °C and 19 °C). After 1 to 6 d of initial illuminance exposure, plates were transferred to darkness (0 lx) at either 25 °C or 19 °C for 14 to 19 d. The x-axis indicates the incubation days for the pre-transfer (pre) and post-transfer (post) stages. The y-axis represents the mean number of perithecia per OTA plate. Data are presented as mean ± SD from four biological replicates.

To dissect the stage-specific light requirement for perithecial production, we performed sequential light-to-dark transfer experiments during the sexual stage (Figure 3 and Figure S3). After a 3 d pre-incubation in darkness (0 lx) at 25 °C, the plates were exposed to four initial illuminance-temperature regimes (5000 lx, 25 °C; 5000 lx, 19 °C; 1000 lx, 25 °C; and 1000 lx, 19 °C) for 1 to 6 d, and then transferred to continuous darkness (0 lx at either 25 °C or 19 °C) for the remaining 14 to 19 d. Perithecia were formed in all eight light-to-dark transfer groups, confirming that early illuminance priming during the mating phase of G10a and G10b is sufficient to support perithecial production even after subsequent continuous darkness. For instance, in the ‘5000 lx, 25 °C → 0 lx, 25 °C’ group, mean yields of perithecia increased progressively from 38 for 1 d to 658 on 6 d of initial illuminance exposure. For all eight transfer treatments, perithecial production exhibited a positive correlation with the duration of initial illuminance exposure. These findings suggested that illuminance priming at the pre-transfer stage promotes perithecial production in a dose-dependent manner.

### 3.3. Early Darkness Suppresses Perithecial Production

**Figure 4:**
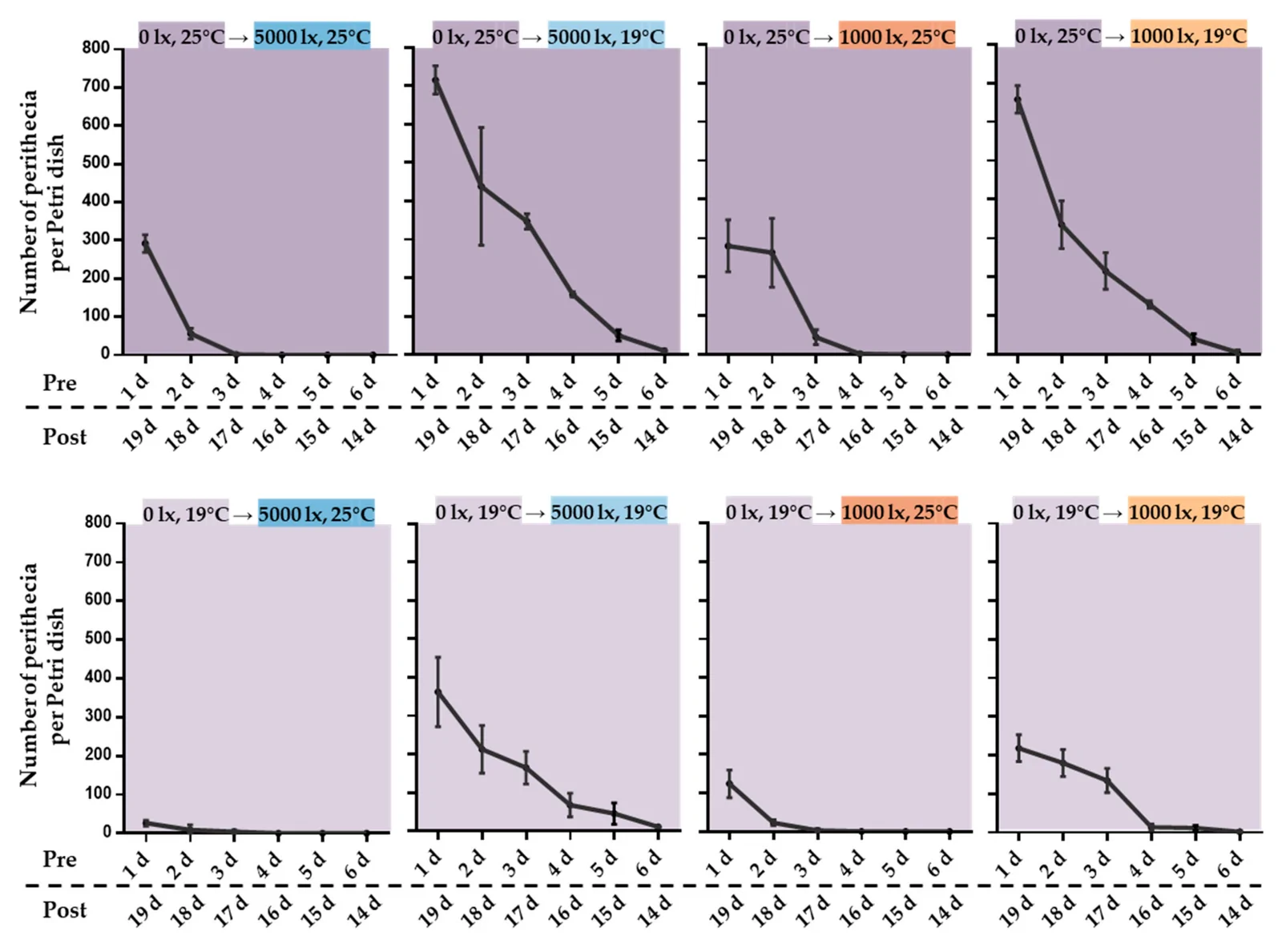
Suppressive effects of initial dark exposure on perithecial production in dark-to-light transfer assays. During the sexual stage, plates were initially maintained in darkness at 25 °C (**upper row**) or 19 °C (**lower row**) for 1 to 6 d, and then transferred to light environments with the indicated illuminance-temperature combinations. The x-axis indicates the incubation days for the pre-transfer stage (pre) and post-transfer stage (post). The y-axis shows the mean number of perithecia per OTA plate. Data are presented as mean ± SD from four biological replicates.

Based on the finding that crossing plates of G10a × G 10b produce no perithecia under constant darkness, we performed dark-to-light transfer assays to test whether subsequent illuminance exposure could rescue fertility in plates subjected to early darkness (Figure 4 and Figure S4). After a 3 d pre-incubation in darkness (0 lx) at 25 °C, plates were cultured in continuous darkness (0 lx) at either 25 °C or 19 °C for 1 to 6 d, and then transferred to light conditions (5000 lx at 25 °C or 19 °C, and 1000 lx at 25 °C or 19 °C) for the remaining 14 to 19 d. The results showed that perithecial production declined progressively with increasing darkness duration across all eight treatment groups. For example, in the “0 lx, 25 °C → 5000 lx, 25 °C” group, mean perithecia yields dropped from 292 at 1 d to 56 at 2 d, reaching 2 at 3 d and 0 at 4 d of initial dark exposure. Across all eight transfer treatments, perithecial production showed a similar negative correlation with the duration of early darkness, and the loss of perithecial production induced by prolonged early dark incubation could not be rescued by subsequent illumination within the experimental timeframe. These findings suggested that early darkness suppresses perithecial production.

### 3.4. Pre-Transfer High Temperature Sustains or Promotes Perithecial Production

**Figure 5:**
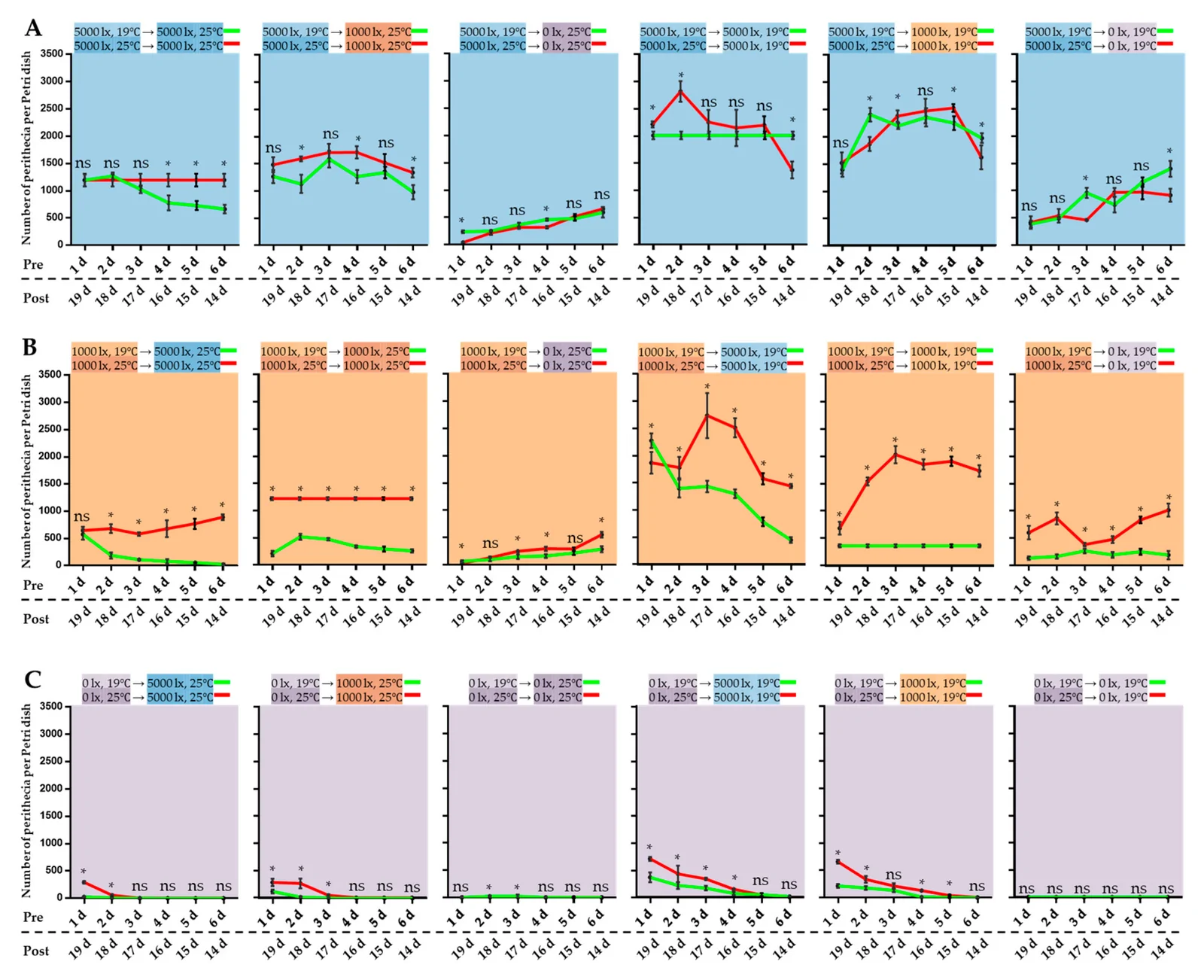
Effects of pre-transfer temperature on perithecial production. Panels (**A**–**C**) present results from full-factorial environmental transfer assays conducted under three initial illuminance levels: (**A**) 5000 lx; (**B**) 1000 lx; and (**C**) 0 lx. Each panel contains six subpanels corresponding to six post-transfer illuminance-temperature combinations (5000 lx, 1000 lx, and 0 lx, each at 25 °C or 19 °C). Green and red lines represent mean yields of perithecia from plates initially cultured at 19 °C and 25 °C, respectively. The x-axis indicates the incubation days for the pre-transfer (pre) and post-transfer (post) stages. The y-axis shows the number of perithecia per OTA plate. Data are presented as mean ± SD from four biological replicates. Statistical significance was determined by the Mann–Whitney U test (Wilcoxon rank-sum test); ns, not significant (*p* > 0.05); *, *p* < 0.05.

To investigate how pre-transfer cultured temperatures affect perithecial production under varying illuminance, we performed a full-factorial environment-transfer assay using G10a × G10b crossing plates (Figure 5). Under high initial illuminance (5000 lx), perithecial yields were comparable between plates pre-incubated at high (25 °C) and low (19 °C) temperatures across all six subsequent environments (Figure 5A and Figure S5). In contrast, under initial low illuminance (1000 lx), perithecial yields were significantly higher in plates pre-incubated at 25 °C than in those pre-incubated at 19 °C (Figure 5B), suggesting that a higher pre-transfer temperature promotes perithecial production under low illuminance. Under initial darkness (0 lx), perithecial yields were substantially suppressed in both temperature groups, but plates initially cultured at 25 °C consistently outperformed those incubated at 19 °C (Figure 5C), further supporting the promotive effect of pre-transfer at high temperature. These results suggested that regardless of the post-transfer temperature, pre-transfer high temperature sustains or promotes perithecial production.

### 3.5. Post-Transfer Low Temperature Sustains or Promotes Perithecial Production

**Figure 6:**
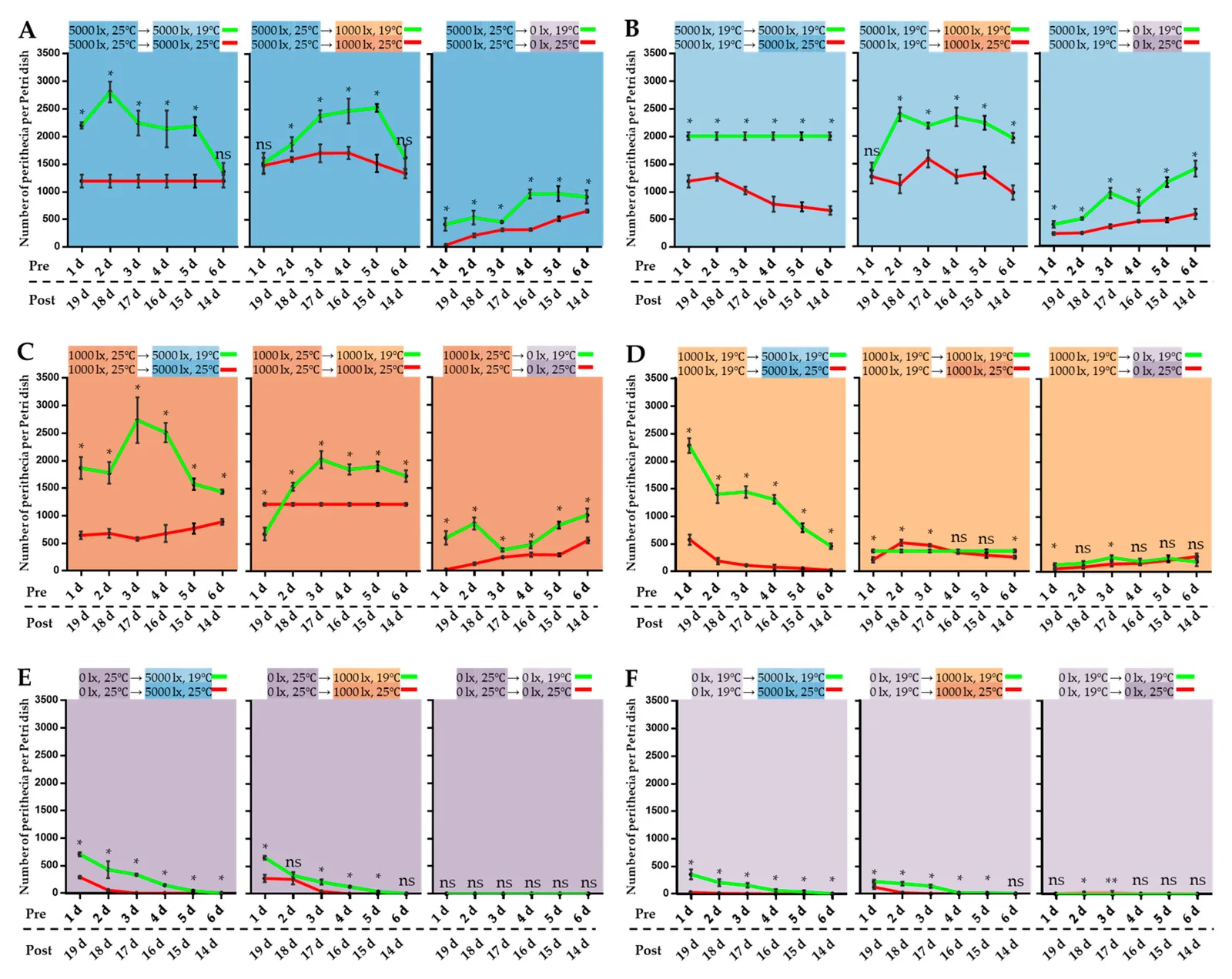
Effect of post-transfer low temperature on perithecial production. Panels (**A**–**F**) display results from full-factorial environmental transfer assays under six distinct initial culture regimes: (**A**) 5000 lx, 25 °C; (**B**) 5000 lx, 19 °C; (**C**) 1000 lx, 25 °C; (**D**) 1000 lx, 19 °C; (**E**) 0 lx, 25 °C; (**F**) 0 lx, 19 °C. Each panel contains three columns corresponding to three post-transfer illuminances (5000 lx, 1000 lx, and 0 lx), within which two post-transfer temperature treatments (19 °C and 25 °C) were compared. Green and red lines represent the post-transfer 19 °C and 25 °C groups, respectively. The x-axis indicates the incubation days for the pre-transfer (pre) and post-transfer (post) stages. The y-axis shows the number of perithecia per Petri dish. Data are presented as mean ± SD from four biological replicates. Statistical significance was determined by the Mann–Whitney U test (Wilcoxon rank-sum test); ns, not significant (*p* > 0.05); *, *p* < 0.05; **, *p* < 0.01.

To investigate how post-transfer temperatures affect perithecial production under different illuminances, we conducted a full-factorial environment transfer experiment and quantified perithecial yields in the G10a × G10b cross (Figure 6). For plates incubated under initial high illuminance (5000 lx; Figure 6A,B), the 19 °C post-transfer condition consistently yielded significantly higher perithecia counts than 25 °C across all three post-transfer illuminance conditions. For plates incubated under initial low illuminance (1000 lx, Figure 6C,D and Figure S6), post-transfer low temperature promoted or sustained perithecial production. Specifically, under the 5000 lx post-transfer condition, perithecial production at 19 °C was significantly higher than at 25 °C. For plates incubated under initial dark conditions (0 lx, Figure 6E,F), perithecial production was drastically reduced in both temperature groups, but the 19 °C group exhibited slightly higher perithecial production than the 25 °C group. These results suggested that post-transfer low temperature either sustains or promotes perithecial production.

### 3.6. High Illuminance Combined with Following Low Temperature Promotes Perithecial Production

**Figure 7:**
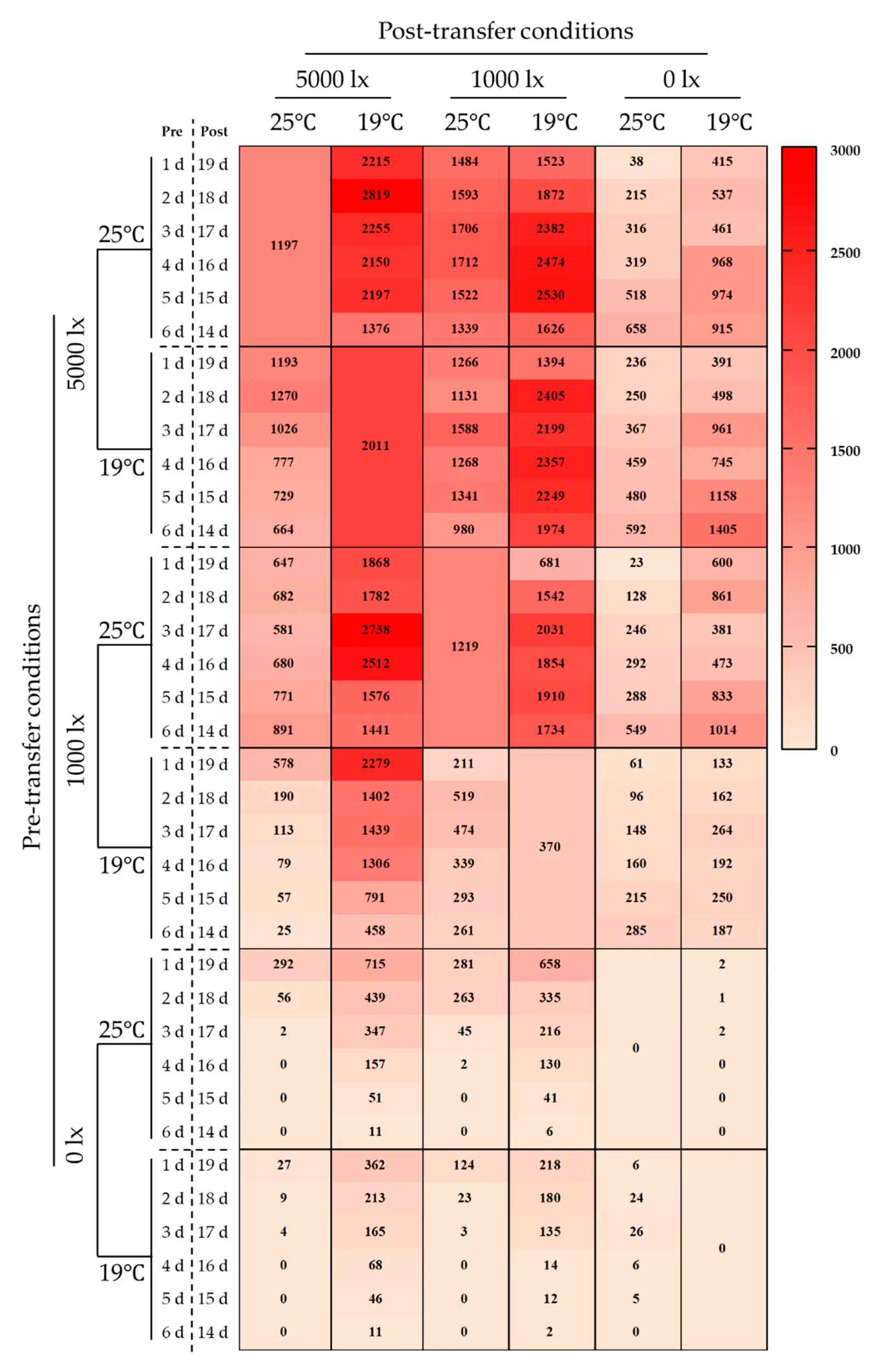
Heatmap of perithecial yields across all combinations with different pre-transfer and post-transfer conditions. Rows represent pre-transfer conditions (illuminance and temperature), and columns represent post-transfer conditions (illuminance and temperature). Dark red indicates higher perithecial yields. Pre, days at pre-transfer incubation stage; post, days at post-transfer incubation stage.

To integrate combinatorial and sequential effects of illuminance and temperature, we constructed a quantitative heatmap of perithecial yields across all 36 treatments over the six pre-transfer days (Figure 7). As shown in Figure 7, pre-transfer conditions played a dominant role in determining the final yields of perithecia. Plates initially cultured at 5000 lx (either 25 °C or 19 °C) retained robust reproductive competence across most post-transfer environments, especially at 19 °C. The maximum yield (2819 per plate) was obtained from plates pre-incubated at 5000 lx and 25 °C for 2 d, followed by transfer to 5000 lx and 19 °C. In contrast, initial dark pre-incubation (0 lx) led to suppression of perithecial production. Plates pre-incubated in darkness for over four days produced fewer than 200 perithecia per plate, even under the optimal post-transfer conditions, and yields dropped to near zero in most environments after six days of initial darkness. These results suggested that high illuminance at the pre-transfer stage combined with low temperature at the post-transfer stage promotes high yields of perithecia.

## 4. Discussion

In this study, we employed a sequential environmental transfer strategy to dissect the individual and combined effects of illuminance and temperature on perithecial production of the green bristlegrass blast fungus *M. oryzae* strains G10a and G10b. Our findings provide alternative insights into the environmental regulation of sexual reproduction and suggest that light should serve as a dominant, stage-specific requirement for perithecial production. Even a single day of light exposure immediately after hyphal contact was sufficient to promote perithecial formation under prolonged subsequent darkness. In contrast, an initial dark incubation results in reductions in perithecial production. These findings suggest that light-dependent commitment to sexual development probably occurs at an early stage of mating in *M. oryzae*, consistent with the conserved developmental program of filamentous ascomycetes, in which light signaling acts upstream of cell–cell recognition and fertilization to initiate sexual differentiation [2,22]. One recent study reported that a light-responsive transcriptional circuit, the MoNFYB-MoRAN1-MoPHO1 pathway, regulates the switch between asexual and sexual development in *M. oryzae*, and that light suppresses the MoRAN1-MoPHO1 module, shifting development toward sexual reproduction [23]. Our results also suggested light-dependent modulation of the effects of temperature on perithecial production, and initial illuminance determined the impact of pre-transfer temperature on the final perithecial yields. At high initial illuminance (5000 lx), pre-transfer temperature had no significant effects on the yield, whereas at low initial illuminance (1000 lx) or in darkness, plates grown at 25 °C consistently produced more perithecia than those at 19 °C. We speculate that under high-illuminance conditions, light signaling might dominate and override temperature-dependent developmental programs; by contrast, under light-limiting conditions, temperature might serve as a major factor governing metabolic activities and sexual differentiation. We also found that low temperature (19 °C) positively promotes perithecial production for the tested G10a and G10b strains. In a recent study, for the rice blast fungus *M. oryzae* strains TH16/TH3, the optimal constant temperature for perithecium formation was 20 °C, and only a few perithecia were produced at 25 °C [18]. Similar phenomena have also been reported in other plant pathogenic fungi, such as *P. aphanis* [5] and *F. graminearum* [6], in which low temperatures promote formation of sexual structures. The underlying mechanism might involve a redirection of metabolic resources from vegetative growth toward sexual development at low temperatures or the activation of temperature-sensitive genes that drive perithecial formation. However, the detailed molecular mechanisms underlying the crosstalk between light and temperature in sexual reproduction remain to be elucidated.

In this study, we tested 36 combinations of illuminance and temperature and obtained the highest-yielding candidate condition for sexual crossing of the green bristlegrass blast fungus *M. oryzae* strains G10a and G10b: following hyphal contact, plates are pre-incubated at 5000 lx and 25 °C for 2 d, then transferred to 5000 lx at 19 °C for 18 d. This regime produced the highest perithecial yield (2819 per OTA plate), about 40% higher than the standard constant culture treatment. For the differences in perithecial yield within the 36 combinations, we speculate that they might be caused by hyphal growth, colony density, or interaction-zone sizes under the combinations of illuminance and temperature. The highest-yielding candidate condition for perithecial production will contribute to research on sexual reproduction, genetic and population analysis, and potential discovery of natural sexual stages in the field. However, our findings are limited to the selected paired *M. oryzae* strains isolated from green bristlegrass, and whether a similar phenomenon occurs in *M. oryzae* strains isolated from other hosts, including rice, foxtail millet, and wheat, remains to be investigated.

In summary, our findings demonstrate that illuminance and temperature act sequentially and interactively to regulate perithecial production of the green bristlegrass blast fungus *M. oryzae* strains G10a and G10b, and that high illuminance combined with a subsequent low temperature promotes perithecial production. Our study provides an alternative perspective on the roles of light and temperature in fungal sexual reproduction.

## Data Availability

The original contributions presented in the study are included in the article/Supplementary Materials; further inquiries can be directed to the corresponding authors.

## Supplementary Materials

The following supporting information can be downloaded at: https://www.mdpi.com/article/10.3390/jof12080564/s1, Figure S1: Fertility assessment and mating-type identification of *M. oryzae* isolates G10a × G10b; Figure S2: Sexual stage of the G10a × G10b crossing; Figure S3: Effect of initial light priming on perithecial production in light-to-dark transfer assays; Figure S4: Effect of initial darkness on perithecial formation in dark-to-light transfer assays; Figure S5: Effect of initial high illuminance duration and pre-transfer temperature on perithecial production; Figure S6: Effect of pre-transfer low illuminance duration and post-transfer temperature on perithecial production; Table S1: Perithecial yields across all combinations of different pre-transfer and post-transfer conditions in the *M. oryzae* G10a × G10b cross.
