# Supplementary material for: High Illuminance and Low Temperature Promote Perithecial Production of the Green Bristlegrass Blast Fungus Magnaporthe oryzae
Source: J Fungi (Basel). 2026 Aug 1;12(8):564. doi: 10.3390/jof12080564 (PMC13514246; doi:10.3390/jof12080564)
Supplement: Supplementary file 1 [file jof-12-00564-s001.zip › jof-4420077-supplementary.pdf]

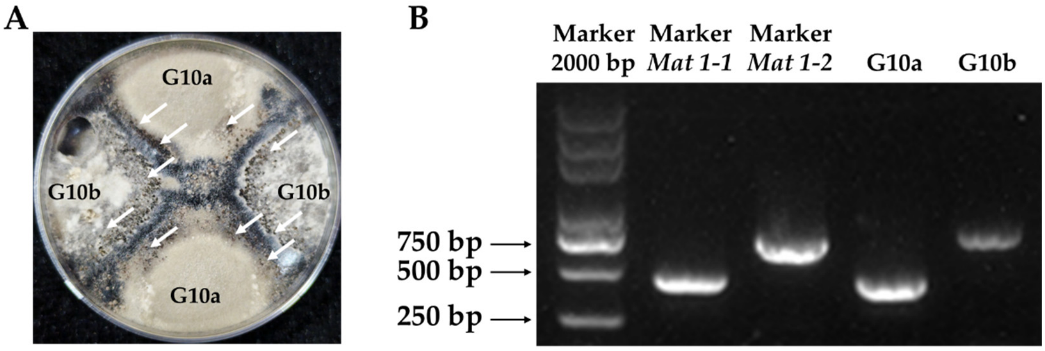

Figure S1. Fertility assessment and mating-type identification of *M. oryzae* isolates G10a × G10b. (**A**) The G10a × G10b cross exhibited high fertility on an OTA plate. White arrows indicate the perithecia. (**B**) Mating-type detection via PCR and gel electrophoresis. G10a and G10b were identified as *MAT1-1* and *MAT1-2*, respectively.

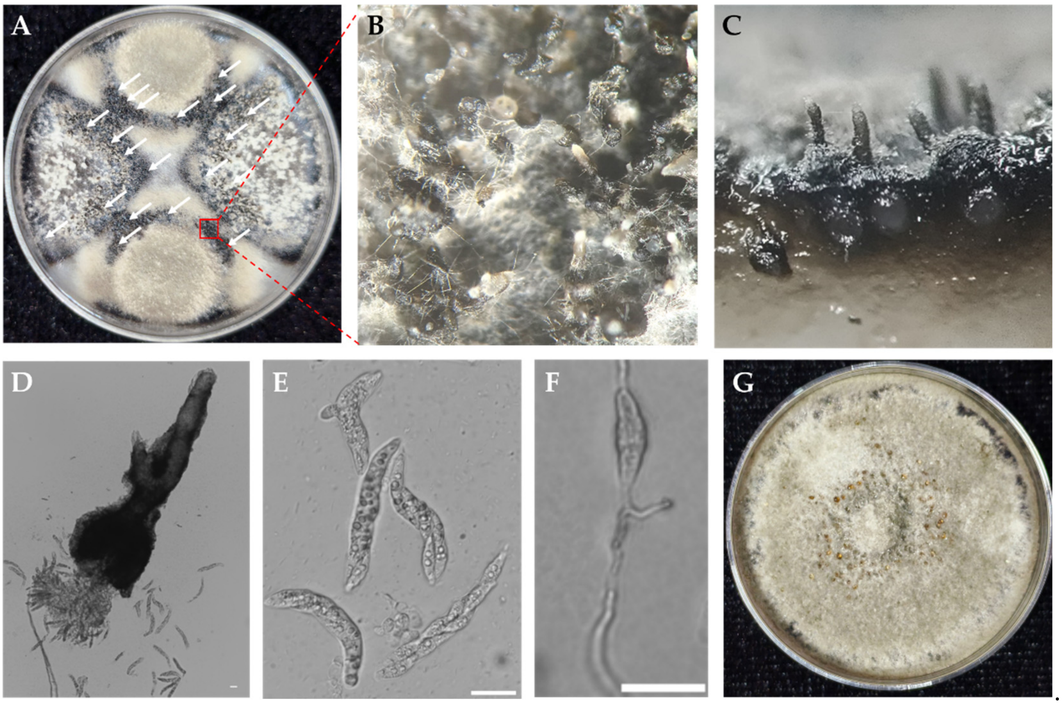

Figure S2. Sexual stage of the G10a × G10b crossing. (**A**) Perithecia produced on an OTA plate incubated under constant 5000 lx at 19 °C for 20 d. (**B**) Top view of the perithecia. (**C**) Side view of the perithecia. (**D**) One perithecium releasing asci. (**E**) Asci with ascospores. (**F**) Germinated ascospores. (**G**) Colony derived from ascospores. Scale bar, 20 μm.

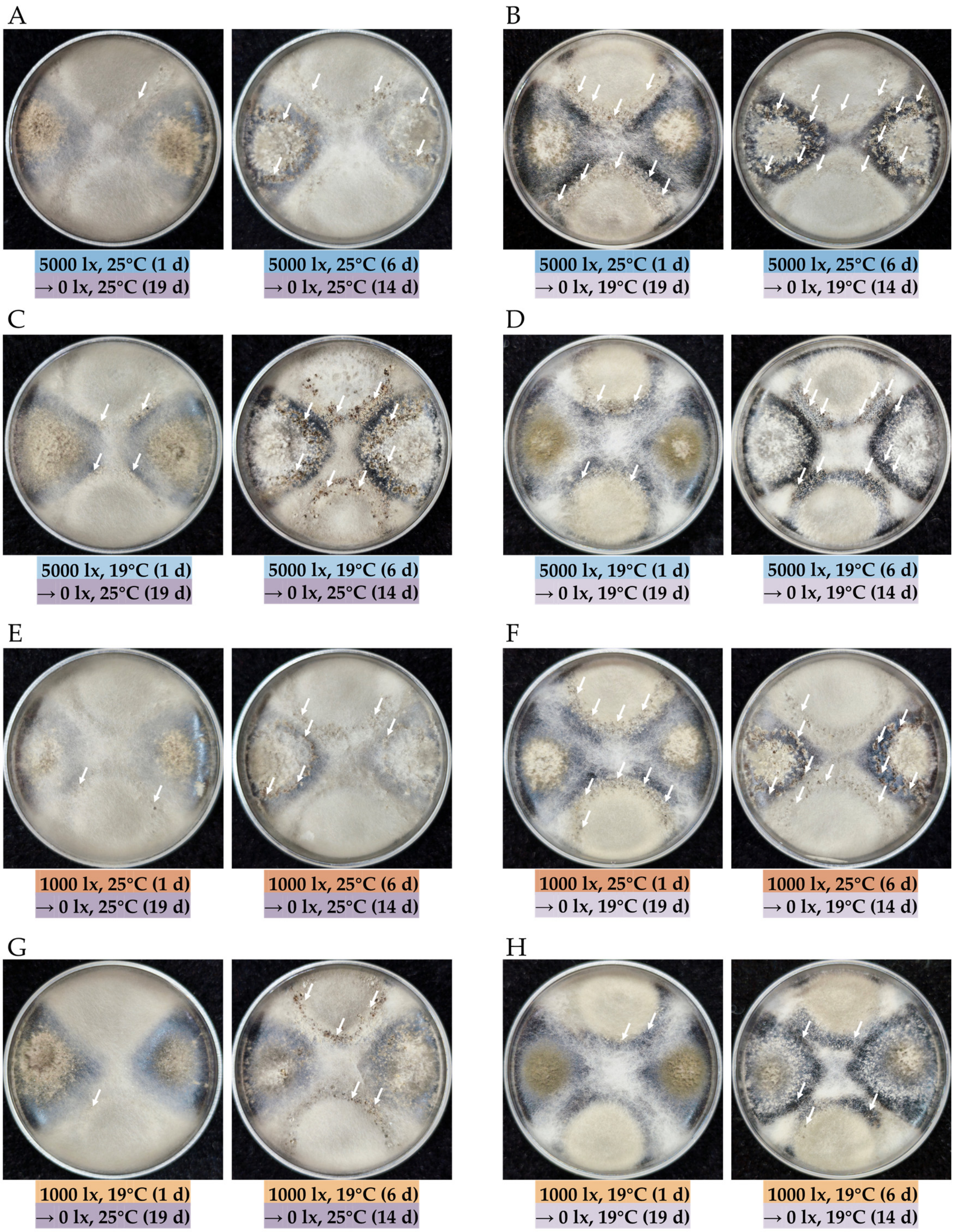

Figure S3. Effect of initial light priming on perithecial production in light-to-dark transfer assays. White arrows indicate perithecia formed at the interaction zones. Each panel displays two representative plates corresponding to treatments with 1 d (**left**) and 6 d (**right**) of initial light exposure, followed by transfer to continuous darkness for 19 d (**left**) and 14 d (**right**), respectively. Panels (**A–D**) and (**E–H**) represent initial illuminance with 5000 lx and 1000 lx, respectively. The text below each image denotes the precise pre-transfer and post-transfer conditions (illuminance, temperature, and duration).

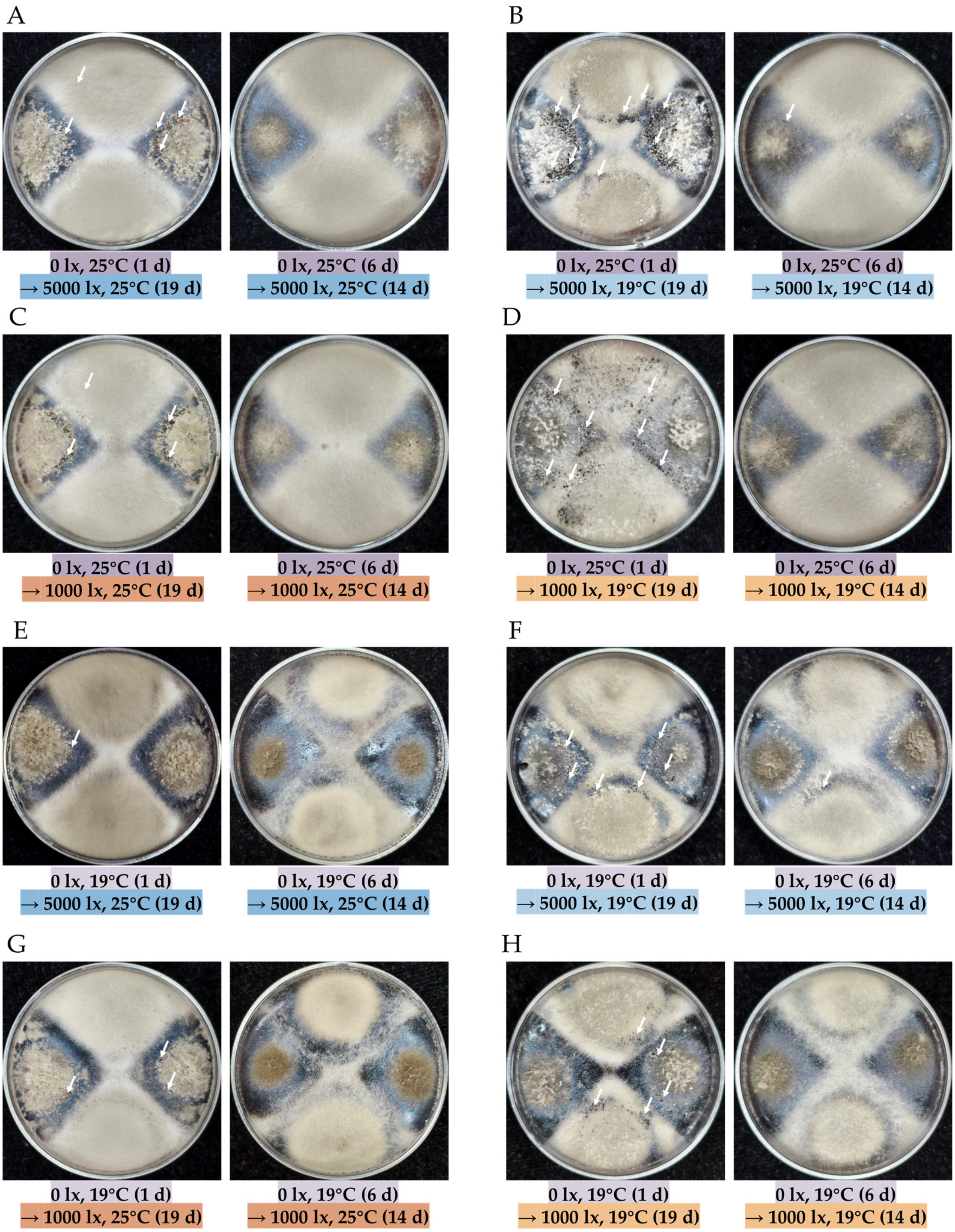

Figure S4. Effect of initial darkness on perithecial formation in dark-to-light transfer assays. White arrows indicate perithecia formed at the interaction zones. Each panel displays two representative plates corresponding to treatments with 1 d (**left**) and 6 d (**right**) of initial darkness, followed by transfer to light exposure for 19 d (**left**) and 14 d (**right**), respectively. Panels (**A–D**) and (**E–H**) represent initial dark incubation temperatures of 25°C and 19°C, respectively. Subsequently, all plates were transferred to post-transfer illuminance with 5000 lx or 1000 lx, in combination with 25°C or 19°C. The text below each image denotes the precise pre-transfer and post-transfer conditions (illuminance, temperature, and duration).

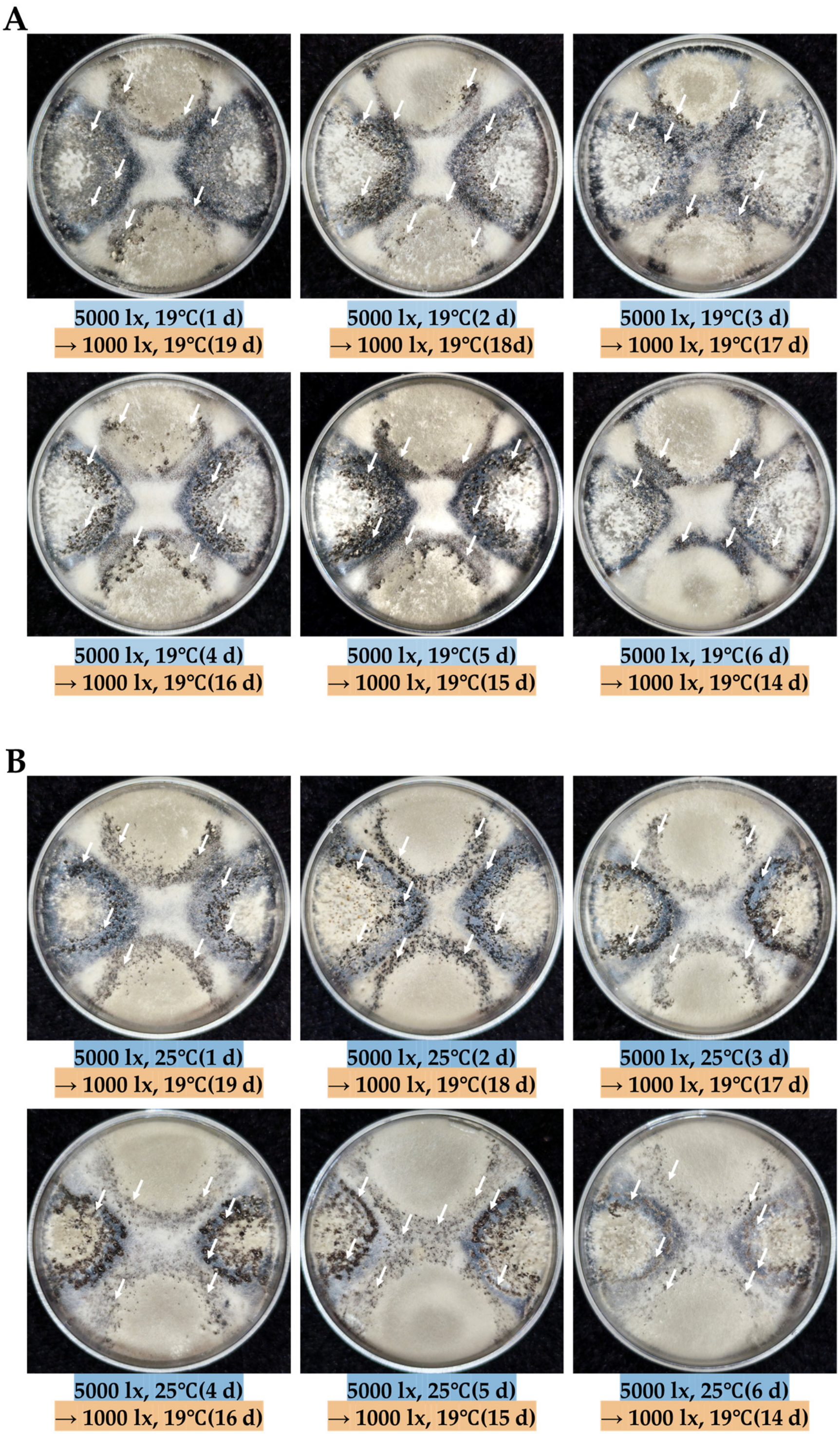

Figure S5. Effect of initial high illuminance duration and pre-transfer temperature on perithecial production. Representative plates showing perithecial formation after 1 d to 6 d of initial incubation at 5000 lx, followed by transfer to 1000 lx, 19°C. (**A**) Plates pre-incubated at 19°C during the pre-transfer stage. (**B**) Plates pre-incubated at 25°C during the pre-transfer stage. White arrows indicate perithecia formed at the interaction zones. The text below each image denotes the precise pre-transfer and post-transfer conditions (illuminance, temperature, and duration).

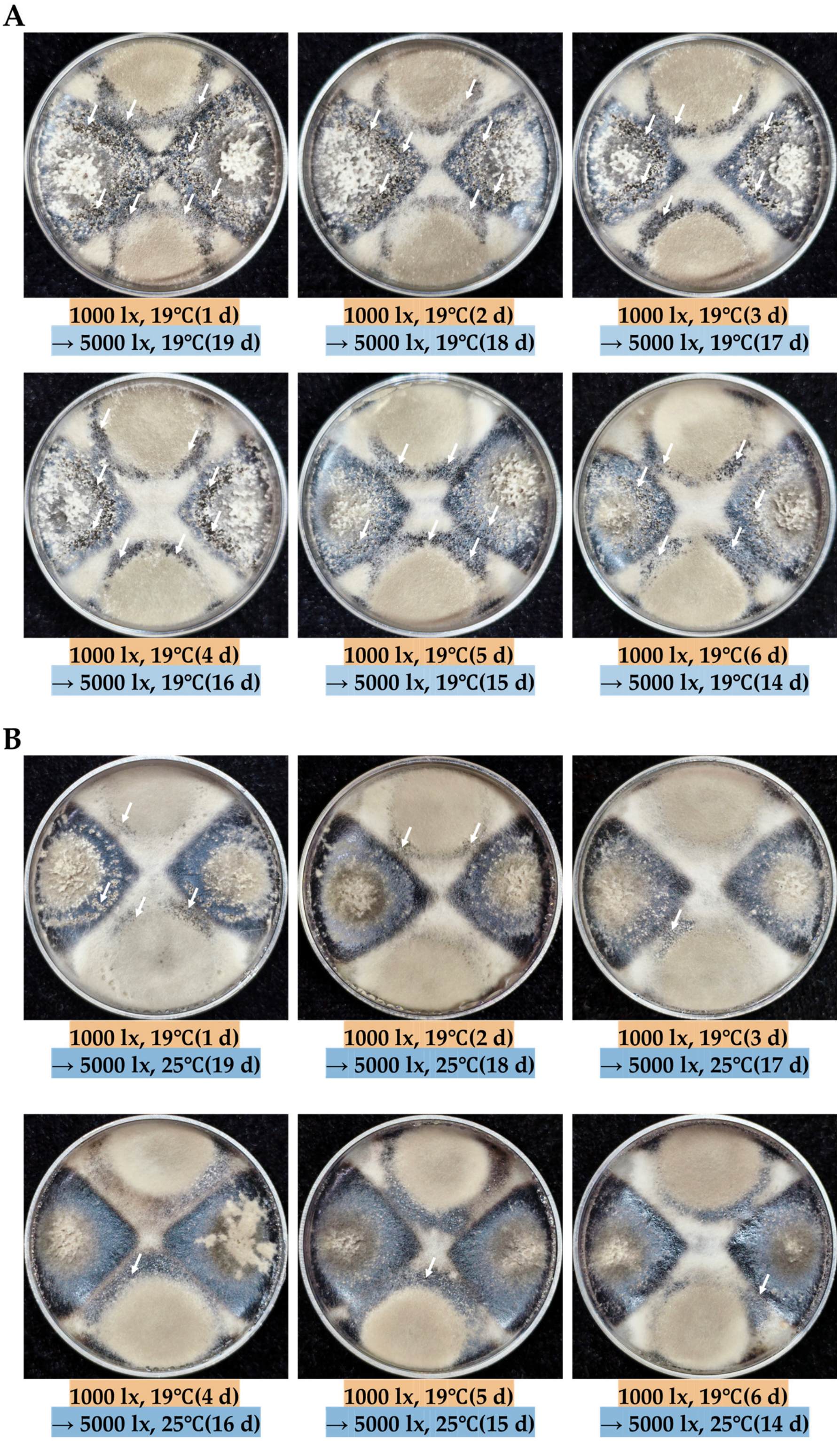

Figure S6. Effect of pre-transfer low illuminance duration and post-transfer temperature on perithecial production. Representative plates showing perithecial formation after initial incubation at 1000 lx and 19°C for 1 to 6 d, followed by transfer to 5000 lx. (**A**) Post-transfer stage maintained at 19°C. (**B**) Post-transfer stage maintained at 25°C. White arrows indicate perithecia formed at the interaction zones. The text below each image denotes the precise pre-transfer and post-transfer conditions (illuminance, temperature, and duration).

Table S1. Perithecial yields across all combinations of different pre-transfer and post-transfer conditions in the *M. oryzae* G10a × G10b cross.

Pre-incubated condition	Pre-transfer condition	duration	Post-transfer conditions					
0 lx, 25° C;	5000 lx, 25° C	Pre → Post	5000 lx, 25° C	5000 lx, 19° C	1000 lx, 25° C	1000 lx, 19° C	0 lx, 25° C	0 lx, 19° C
		1 d → 19 d		2152; 2240; 2186; 2281	1342; 1388; 1607; 1598	1705; 1649; 1281; 1457	26; 56; 38; 32	390; 265; 475; 529
		2 d → 18 d		2590; 3032; 2895; 2760	1656; 1588; 1546; 1580	1776; 2000; 1748; 1962	170; 204; 246; 240	448; 454; 529; 717
	124 plates	3 d → 17 d	1072<br>1352<br>1186<br>1180	2309; 2525; 2202; 1984	1804; 1861; 1662; 1498	2519; 2380; 2267; 2360	299; 327; 291; 345	458; 478; 461; 445
		4 d → 16 d		2043; 1727; 2360; 2469	1600; 1798; 1630; 1818	2808; 2356; 2396; 2337	341; 309; 329; 295	990; 848; 998; 1034
		5 d → 15 d		2056; 2129; 2441; 2160	1445; 1376; 1528; 1740	2580; 2592; 2509; 2439	493; 477; 523; 578	879; 838; 1097; 1082
		6 d → 14 d		1491; 1235; 1247; 1530	1362; 1233; 1313; 1448	1767; 1703; 1733; 1301	694; 659; 621; 659	863; 778; 964; 1054
	5000 lx, 19° C	Pre → Post	5000 lx, 25° C	5000 lx, 19° C	1000 lx, 25° C	1000 lx, 19° C	0 lx, 25° C	0 lx, 19° C
		1 d → 19 d	1229; 1056; 1165; 1322;		1182; 1146; 1347; 1390	1487; 1204; 1400; 1483	250; 218; 214; 263	416; 465; 337; 347
		2 d → 18 d	1220; 1269; 1364; 1228		942; 1257; 1039; 1287	2504; 2273; 2324; 2518	254; 252; 262; 232	503; 487; 477; 523
	124 plates	3 d → 17 d	1067; 930; 1089; 1016	1927<br>2024<br>1994<br>2099	1800; 1604; 1487; 1461	2114; 2228; 2227; 2225	390; 398; 313; 365	931; 978; 856; 1079
		4 d → 16 d	573; 821; 879; 835		1368; 1179; 1378; 1147	2488; 2448; 2373; 2117	444; 487; 462; 442	862; 874; 638; 604
		5 d → 15 d	695; 853; 694; 674		1410; 1196; 1434; 1322	2379; 2098; 2326; 2192	513; 489; 499; 420	1150; 1063; 1275; 1144
		6 d → 14 d	655; 558; 695; 746		972; 832; 963; 1151	2006; 1984; 2057; 1849	574; 479; 606; 709	1390; 1317; 1556; 1295
	1000 lx, 25° C	Pre → Post	5000 lx, 25° C	5000 lx, 19° C	1000 lx, 25° C	1000 lx, 19° C	0 lx, 25° C	0 lx, 19° C
		1 d → 19 d	632; 745; 632; 578	1986; 1917; 1992; 1576		804; 571; 756; 592	32; 18; 32; 8	487; 509; 744; 661
		2 d → 18 d	786; 678; 677; 588	1742; 1607; 1717; 2061		1481; 1618; 1477; 1592	141; 152; 119; 99	924; 981; 791; 749
	124 plates	3 d → 17 d	545; 612; 561; 604	2269; 2893; 2560; 3228	1227<br>1190<br>1256<br>1204	2008; 2242; 2018; 1857	244; 235; 247; 257	372; 368; 429; 353
		4 d → 16 d	517; 788; 834; 579	2760; 2460; 2350; 2477;		1771; 1940; 1930; 1774	233; 316; 301; 317	551; 491; 416; 432
		5 d → 15 d	691; 691; 875; 827	1596; 1440; 1578; 1691		1810; 1869; 1978; 1984	303; 269; 321; 260	915; 837; 812; 766
		6 d → 14 d	903; 913; 934; 814	1465; 1396; 1479; 1422		1770; 1841; 1590; 1733	626; 519; 539; 512	847; 1011; 1079; 1118
744 plates	1000 lx, 19° C	Pre → Post	5000 lx, 25° C	5000 lx, 19° C	1000 lx, 25° C	1000 lx, 19° C	0 lx, 25° C	0 lx, 19° C
		1 d → 19 d	709; 487; 578; 539	2289; 2437; 2109; 2281	224; 203; 147; 269		93; 55; 55; 40	164; 154; 111; 101
		2 d → 18 d	137; 214; 260; 149	1475; 1507; 1159; 1467	574; 552; 446; 505		109; 137; 81; 57	123; 131; 210; 184
	124 plates	3 d → 17 d	117; 129; 101; 103	1578; 1362; 1460; 1356	451; 499; 457; 489	382<br>346<br>404<br>349	89; 139; 194; 170	198; 280; 290; 287
		4 d → 16 d	46; 63; 60; 147	1360; 1293; 1372; 1198	321; 339; 369; 325		150; 212; 141; 135	227; 244; 175; 123
		5 d → 15 d	52; 57; 67; 53	699; 766; 893; 804	289; 245; 356; 280		204; 184; 267; 206	196; 311; 210; 283
		6 d → 14 d	8; 14; 46; 32	430; 414; 533; 453	268; 266; 291; 218		327; 222; 336; 256	123; 115; 261; 248
	0 lx, 25° C	Pre → Post	5000 lx, 25° C	5000 lx, 19° C	1000 lx, 25° C	1000 lx, 19° C	0 lx, 25° C	0 lx, 19° C
		1 d → 19 d	295; 279; 271; 323	756; 689; 679; 737	259; 376; 220; 267	635; 629; 707; 661		2; 0; 2; 2
		2 d → 18 d	75; 42; 57; 51	574; 315; 297; 568	178; 285; 378; 210	422; 283; 333; 303		0; 0; 4; 0
	124 plates	3 d → 17 d	6; 0; 0; 0	370; 356; 325; 337	73; 40; 28; 40	271; 224; 212; 156	0<br>0<br>0<br>0	2; 4; 0; 0
		4 d → 16 d	0; 0; 0; 0	160; 164; 149; 154	0; 0; 8; 0	121; 127; 127; 143		0; 0; 0; 0
		5 d → 15 d	0; 0; 0; 0	34; 55; 67; 46	0; 0; 0; 0	30; 50; 54; 28		0; 0; 0; 0
		6 d → 14 d	0; 0; 0; 0	10; 16; 6; 10	0; 0; 0; 0	0; 0; 14; 8		0; 0; 0; 0
	0 lx, 19° C	Pre → Post	5000 lx, 25° C	5000 lx, 19° C	1000 lx, 25° C	1000 lx, 19° C	0 lx, 25° C	0 lx, 19° C
		1 d → 19 d	26; 34; 30; 16	360; 241; 457; 390	117; 123; 172; 85	226; 178; 208; 261	22; 0; 0; 0	
		2 d → 18 d	0; 7; 28; 0	147; 244; 178; 283	26; 26; 12; 28	149; 224; 155; 192	10; 34; 32; 20	
	124 plates	3 d → 17 d	10; 6; 0; 0	210; 160; 109; 182	2; 10; 0; 0	131; 179; 107; 121	29; 11; 48; 15	0<br>0<br>0<br>0
		4 d → 16 d	0; 0; 0; 0	87; 22; 79; 82	0; 0; 0; 0	4; 8; 20; 22	0; 0; 12; 10	
		5 d → 15 d	0; 0; 0; 0	26; 28; 59; 69	0; 0; 0; 0	2; 12; 14; 18	0; 0; 20; 0	
		6 d → 14 d	0; 0; 0; 0	10; 10; 10; 14	0; 0; 0; 0	0; 0; 6; 0	0; 0; 0; 0	
